# Secondhand smoke exposure assessment and counseling in the Chinese pediatric setting: a qualitative study

**DOI:** 10.1186/1471-2431-14-266

**Published:** 2014-10-15

**Authors:** Jing Liao, Abu S Abdullah, Guangmin Nong, Kaiyong Huang, Longde Lin, Zhenyu Ma, Li Yang, Zhiyong Zhang, Jonathan P Winickoff

**Affiliations:** Department of Pediatrics, The First Affiliated Hospital of Guangxi Medical University, Nanning, Guangxi 530021 China; Global Health Initiative, Duke Kunshan University, Kunshan, Jiangsu Province 215347 China; Duke Global Health Institute, Duke University, 310 Trent Drive, Durham, NC 27710 USA; School of Public Health, Guangxi Medical University, 22 Shuangyong Road, Nanning, Guangxi 530021 China; Boston University School of Medicine, Boston Medical Center, Boston, MA USA; MGH Center for Child and Adolescent Health Research and Policy, Harvard Medical School, Boston, USA

**Keywords:** Secondhand smoke, Pediatric setting, Healthcare workers, Counseling, Chinese, Tobacco control, Qualitative study

## Abstract

**Background:**

Assisting smoking parents to quit smoking and eliminating the secondhand smoke (SHS) exposure of their children is a global health priority. Engaging healthcare workers in developing countries to address this priority has been a challenge. This study intends to explore issues around current practice related to SHS exposure assessment and counseling and identify barriers to SHS exposure reduction counseling in the Chinese pediatric setting.

**Methods:**

We conducted qualitative interviews (11 focus groups discussions (FGDs) with pediatricians, 6 FGDs with pediatric nurses and 11 in-depth interviews (IDIs) with hospital administrators) among 101 health-care professionals (HCP) from 5 hospitals in four major cities of Guangxi Province, China. All FGDs/ IDIs were audio recorded and analysed thematically.

**Results:**

The findings suggest that few Chinese pediatricians routinely address the SHS exposure of children in their usual practice. All HCPs felt the need for clinical interventions to promote SHS exposure reduction for children. Primary barriers to SHS exposure reduction counseling in the Chinese pediatric setting included: lack of skills and training in tobacco use reduction and cessation counseling; time constraints and heavy workloads, uncertainty about the usefulness of smoking cessation interventions and lack of hospital-wide systems requiring pediatricians to record tobacco use or SHS exposure information. Ideas for overcoming these barriers were building capacity of pediatricians, collaboration with international organization to initiate training, engaging top level leaders in the effort and ensuring financial resources to support the program.

**Conclusions:**

This study among hospital administrators and service providers in China demonstrated a high level of interest in delivering SHS exposure reduction interventions in the pediatric setting. The findings can inform the creation and delivery of clinical interventions in China to promote SHS exposure reduction to children in the pediatric setting.

**Electronic supplementary material:**

The online version of this article (doi:10.1186/1471-2431-14-266) contains supplementary material, which is available to authorized users.

## Background

The health consequences of exposure to secondhand smoke(SHS)also known as tobacco smoke pollution (TSP) are now well accepted [1, 2]. The high prevalence of smoking in many developing countries results in more exposure to SHS among non-smokers and children [1]. As the world’s largest producer and consumer of tobacco [3, 4] about one-third of the world’s tobacco is smoked in China [5]. With over 350 million smokers, China has 740 million non-smokers passively exposed to SHS, including 180 million children under the age of 15 [6, 7]. Children are exposed to higher levels of SHS than adults because children are often unable to change the circumstances that lead to their exposure [8, 9]. In China, proposed workplace legislation may protect adult non-smokers once implemented, but fails to protect children from exposure to SHS in their own homes. The SHS exposure of children due to parental tobacco use is a prevalent health issue in China [5, 10] and is associated with many health conditions [11–14]. The Chinese health care system could be a key channel for delivering tobacco control interventions to parents of young children [15]. Educational interventions delivered by trusted health-care professionals will capitalize on the potential of the teachable moment to enhance parent awareness and inspire parents to eliminate SHS exposure of their family members [11, 15, 16]. Researchers in the United States developed the Clinical Effort Against Secondhand Smoke Exposure (CEASE) a training and dissemination program for pediatricians and office staff [17] which was effective in engaging pediatricians in tobacco control efforts and promoting smoking cessation among parents who smoke [18]. However, similar initiative is not available in China or other developing countries.

To explore issues around current practice related to SHS exposure assessment and counseling and identify barriers to SHS exposure reduction counseling in the Chinese pediatric setting, we conducted a qualitative study among Chinese health-care professionals including pediatricians, pediatric nurses, and hospital administrators. Questions to be explored include: what do Chinese pediatric staff think about development of a parental tobacco control intervention in pediatric setting, how much do Chinese pediatric staff know about SHS assessment and counseling, what are the barriers to SHS exposure reduction counseling in the Chinese pediatric setting.

## Methods

### Sample and settings

Participants were (a) hospital administrators (i.e. president and/or director of department of pediatrics) and (b) pediatric clinical service providers (i.e. pediatricians and pediatric nurses). The settings included six purposively selected hospitals from four major cities in Guangxi province and included three grade III hospitals (First Affiliated Hospital of Guangxi Medical University (Nanning), Maternal and Child Health Hospital (Nanning), Affiliated Hospital of Guilin Medical University (Guilin)), two grade II hospitals (Qinzhou Maternal and Child Health Center (Qinzhou), Liuzhou Maternal and Child Health Center (Liuzhou)) and one community health center (grade 1, Zhuxi Community Health Center (Nanning)) We conveniently selected these hospitals as there are pediatric department in each of these hospitals. Hospital systems in China follow a grading system. The higher the grade, the larger the hospital and the more sophisticated the facility is. Level three hospitals are general or comprehensive hospital at national, provincial or city level (>500 beds); level two hospitals are hospitals of medium size at city, county and district level (between 100–500 beds); and level one hospitals are the township hospitals (<100 beds). The size and characteristics of the pediatric departments and the patient population within different levels of hospital differs according to the grade of the hospital.

### Procedures

We conducted one-to-one in depth interviews (IDIs) with the hospital administrators and focus groups discussions (FGDs) with pediatric clinical service providers during April-May 2013. Participants were recruited through the hospital liaisons in each participating hospital. The liaison person, a senior pediatrician, was provided with the verbal and written background information of the study and the kind of people we were looking for to participate in the exploratory study. The liaison person then invited potential subjects for voluntary participation and arranged a schedule for IDI or FGD.

### Data collection

All of the FGDs/IDIs were conducted in Mandarin Chinese using a semi-structured interview guide and audio recorded [19]. A FGD guide was developed with reference to the research team’s earlier work [19] and pilot tested with one hospital administrator, four pediatricians and four pediatric nurses resulting in minor changes. The guide included questions and queries on the following themes: attitudes towards SHS, knowledge of children’s health in relation to SHS exposure, current practice related to SHS assessment and counseling in the Chinese pediatric setting, perceived barriers to providing SHS exposure reduction counseling, and suggestions for overcoming the potential barriers.

Four interviewers conducted all the FGDs and IDIs. Interviewers were graduate students at the School of Public Health of Guangxi Medical University, and attended a 2-day training course on qualitative research methods and tobacco use reduction research. The training also included a session on the ethical aspects of human subject research. To collect data, two worked as a team; one moderated the FGD/IDI and the other took detailed notes and recorded the session with a digital voice recorder (with permission from the participants). All FGDs were held at the hospital in a private meeting room, and lasted for approximately 90 minutes. The sessions started with the moderator explaining the purpose of the group discussion and assuring confidentiality of the data collected for the research project. The IDIs were conducted in a location convenient for the participants and took about 60 minutes. To compensate for their time, each participant was given a cash amount of RMB 100 ($15).

Written informed consent was obtained from each participant. The study was approved by the Ethics Committee of the Guangxi Medical University (No: IRB-Int-2013 (315–1).

### Analyses

The interviewers discussed and summarized the content of each IDI or FGD and reviewed the notes taken immediately after the interview or discussion. These debriefings were useful i) to identify most important themes and ideas and ii) to assess the need for any modification in the subsequent IDI or FGD. The audio recordings were reviewed and transcribed for each group or IDI. Two members of the research team coded each transcript independently, with discrepancies resolved through consensus. The process of coding involved identifying key themes and marking these out on the transcripts [20]. All additional notes taken during the course of the focus groups and interviews were examined to identify various themes presented in these qualitative discussions. All the analysis was conducted in English. All the transcripts was translated from Mandarin to English and then back translated.

## Results

Seventeen FGDs (five FGDs with pediatricians who smoke, six FGDs with pediatricians who never smoked, six FGDs with pediatric nurses; 4–6 participants in each FGD) and 11 IDIs with hospital administrators were conducted among 101 participants from 6 hospitals in four major cities of Guangxi Province, China. This included 59 pediatricians (26 smokers, 33 non-smokers), 31 pediatric nurses (all non-smokers), and 11 hospital administrators (1 smokers, 10 non-smokers) (Table 1).

**Table 1:** ***Characteristics of participants*** **(n=101)**

Characteristics	Focus group discussions	In-depth interviews
	(FGDs) (17 FGDs; n=90)	(IDIs) (n=11)
Gender	35 males, 55 females	9 males, 2 females
Age (mean ± SD)	31.49 ± 3.26 years	50.82 ± 4.75 years
	range (27–38)	range (42–58)
Pediatricians	59	
Smokers	26	
Non-smokers	33	
Pediatric nurses	31	
Smokers	0	
Non-smokers	31	
Hospital administrators		11
Smoker		1
Non-smokers		10

Four main themes relating to SHS assessment and counseling emerged: knowledge of and attitude towards children’s SHS exposure; current practice related to SHS assessment and counseling; barriers to provide SHS exposure reduction counseling; and views and suggestions for overcoming these barriers. These themes are described below supplemented by participants’ statements on key themes provided in Additional file 1 as *supplemental information*.

### Knowledge of and attitude towards children’s SHS exposure

Almost all (100/101) participants believed that exposure to SHS is harmful to children’s health and expressed opposition to SHS exposure among children.

*“Exposure to SHS is harmful to children’s health. Parents should protect their children from SHS exposure.”* (one participant shouted)

Regarding the specific health risk to children from SHS exposure, the majority (79/101) of participants believed that SHS exposure puts children at risk for asthma, flu, bronchiolitis, and pneumonia with some exceptions. Several participants argued against the health consequences of SHS exposure to these health conditions.

### Current practice related to SHS assessment and counseling in the Chinese pediatric setting

Most (50/59) of the pediatricians reported that they did not routinely enquire about children’s SHS exposure and provide smoking cessation counseling to smoking parents in their practice. They would ask about patients’ SHS exposure sometimes when they treated patients with respiratory diseases, such as asthma. Many healthcare providers thought that it is only relevant to talk with parents about smoking and SHS, if the child’s disease is associated with smoking.

Some (20/59) pediatricians and pediatric nurses (18/31) reportedly would ask about children’s SHS exposure in their practice occasionally, but few of them felt confident to provide smoking cessation counseling to smoking parents.

*“It is not a part of my job and it is not required in our hospital.”* (Few pediatricians and few nurses).

None of the participants reported documenting and monitoring SHS exposure of children and parental smoking systematically in pediatric settings.

### Barriers to SHS exposure reduction counseling in the Chinese pediatric setting

When asked about barriers to providing SHS exposure reduction counseling, the following responses emerged:

#### Lack of skills and training in tobacco control and SHS exposure reduction counseling

Some participants stated they lacked skills related to tobacco control and SHS exposure reduction counseling, which hindered them from providing SHS exposure reduction counseling to smoking parents.

*“My current knowledge and skills related to tobacco control and SHS exposure reduction counseling are not sufficient for helping patients to stop smoking.”* (one pediatricians who never smoke)

Overall, the majority of pediatricians (49/59) and pediatric nurses (21/31) reportedly did not receive any formal training in tobacco control and SHS exposure reduction counseling, and they did not know any smoking cessation clinic or quitline in their city.

#### Time constraints and heavy workloads

Many participants believed that talking to smoking parents about tobacco control and SHS exposure reduction during routine visits was important; however, they felt they lacked time.

More than three quarters of pediatricians (52/59) and almost all the pediatric nurses (26/31) expressed that they hardly spent extra time to enquire about children’s SHS exposure and provide smoking cessation counseling to smoking parents in their practice, because of time constraints and their heavy workloads.

#### Not convinced that smoking cessation and SHS exposure reduction counseling would be effective

Several (7/59) pediatricians thought that smoking cessation and SHS exposure reduction counseling may not be effective in helping smoking parents quit smoking. However, few knew about the efficacy of counseling and medications.

*“I am not convinced of the efficacy of nicotine replacement therapy, such as nicotine patch or gum.”* (One pediatrician who smokes)

#### Lack of system-wide approach that requires providing SHS exposure reduction and smoking cessation counseling

A number of participants felt the need for a systematic approach within the hospital that will require them to assess smoking or SHS exposure status of their patients as a first step. They would then provide smoking cessation or SHS exposure reduction counseling, if the hospital system allows them to do so.

*“I do not ask my patients anything about smoking or SHS exposure. It is because, I myself am a smoker. Also it is not required by my job.”* (A pediatrician who smokes)

### Views and suggestions for overcoming the potential barriers

When asked about suggestions for overcoming these barriers, many participants (79/101, 78%) expressed that they were willing to enhance collaboration with international organization such as World Health Organization (WHO) and the American Academy of Pediatrics (AAP) to promote smoking cessation and SHS exposure reduction in China.

Several participants (four hospital administrators and twelve pediatricians) suggested that the tobacco control and SHS exposure reduction intervention ought to be adapted to the Chinese pediatric setting following the Chinese healthcare system and cultural norms. Some participants (34/101, 34%) expressed interest in receiving formal training in tobacco control and SHS exposure reduction counseling to improve their smoking cessation and SHS exposure reduction counseling skills. Some of them (15/101, 15%) even suggested organizing the trainings in a flexible manner for better participation, including organizing the training during weekday evenings or weekend mornings with arrangements for breakfast or lunch or dinner, and offering the same session in multiple occasion to allow each to participate.

Some participants suggested the provision of a certificate to attend the training course.

In order not to increase pediatricians’ workloads and not to disrupt pediatric office operations, a few participants (18/101, 18%) suggested that additional persons should be specially assigned to provide SHS exposure reduction counseling to smoking parents during routine children’s clinic visits.

Financial difficulties to deliver smoking cessation programs were raised by few hospital administrators. In the absence of national funding, they believed funding could be available from international agencies (i.e. WHO or AAP) to initiate some pilot programs.

Few participants thought that commitment and support from hospital leaders are important to initiate any system change and capacity building effort.

### Smokers versus non-smoker participants

We have observed a difference in views between smokers and non-smokers. Most non-smokers discussed health consequences of SHS exposure to children, were in favor of adopting measures to address children’s exposure to SHS and how such measures could be implemented; however, few smokers were defensive, tried to argue against the effort to come up with ideas such as, wider acceptance of tobacco use in the Chinese society with no serious health problems among certain leaders, and to explain how any intervention would not be feasible in the Chinese society or healthcare system. However, in most situations these smokers would compromise and would agree with the nonsmokers.

## Discussion

In seeking to inform the development of a culturally appropriate, feasible, and systematic strategy for addressing SHS exposure reduction among children in the pediatric setting, we gathered insights from medical service providers and hospital administrators in China. The inclusion of both doctors and nurses, and hospital leaders in the study generated balanced information addressing both a practice-related and policy-oriented perspective. This balanced information could guide the development of a clinical intervention (i.e. CEASE progarm) to be implemented in the Chinese pediatric setting.

In this study, most participants believed that exposure to SHS is harmful to children’s health and expressed opposition to SHS exposure among children, but only few of them routinely addressed the SHS exposure of children in the usual practice. Similar to the findings in other studies [21], some pediatricians would enquire about children’s SHS exposure status only if the child’s disease was associated with smoking and passive smoking. The lack of clinical attention towards tobacco exposure reduction is not surprising given the fact that there are no guidelines or recommendations from professional societies such as the Chinese Pediatric Society or the Chinese Academy of Medicine. In the US, both the American Academy of Pediatrics and the American Academy of Family Physicians recommend that practitioners assess their patients’ exposure to SHS and provide exposure reduction counseling [22, 23].

We found that many pediatricians lacked adequate training and skills related to tobacco control and SHS exposure reduction counseling, and many expressed the need for training. At the same time, the efficacy of smoking cessation counseling or medications and SHS exposure reduction counseling was not clear to many participants, supporting the findings of another study among Chinese physicians [24]. These misconceptions reflect the fact that more formal trainings on SHS exposure reduction and smoking cessation counseling among pediatricians are needed. In the United States, training pediatricians and office staff systematically on the CEASE program has led to an increase in the provision of cessation assistance [17, 18]. Developing a training model to address SHS exposure of children and parental smoking based on the strategies of CEASE trainings and implementation in the United States may help to enhance SHS exposure reduction counseling in Chinese pediatric settings.

In this study, many participants blamed limited time and workload in hindering them to provide SHS exposure reduction counseling to smoking parents. However, evidence from our CEASE program in the USA showed that the smoking cessation support intervention adds only about 30 seconds to 3 minutes to a child’s clinic visit [18]. Suggestions made by a few participants to involve additional personnel specially assigned to provide SHS exposure reduction counseling to smoking parents during routine children’s clinic visits is useful. In the USA, smoking parents are referred to State Quitlines [25] or other available smoking cessation programs [26] by the pediatricians or nurses. In the absence of such program in China, hiring of additional personnel to provide cessation counseling would have great public health impact and should be explored for feasibility.

The findings show that the absence of any policies within the hospital requiring physicians to ask about smoking or SHS exposure is a major barrier to promote tobacco use reduction and cessation. At present, it is not a requirement to record smoking status in the patient’s medical record. Making system-wide changes within the hospital that will require recording smoking or SHS exposure status as a vital sign and then delivering appropriate interventions based on the resources available within the hospital would have an impact. Because tobacco dependence is a disease that needs clinical attention [2], hospitals should consider establishing smoking cessation clinics [27], Quitlines [28], and other targeted programs [15, 29] to ensure the delivery of comprehensive clinical services.

Strengths of this qualitative study include attention to an important issue in the Chinese pediatric clinical setting, recruitment of subjects from different cities, inclusion of both hospital administrators and service providers, inclusion of both smokers and non-smokers in the FGD/IDI, and the use of multiple focus groups. A further strength was the use of open ended questions so that a variety of themes could emerge. There are several limitations. First, the findings may not be representative of the whole pediatric staff in China given that this study took place in the pediatric setting of one region (Guangxi province) of China. Second, there might be selection bias of subjects as those selected were based on their availability and their willingness to participate in the tobacco control research. Third, none of the nurses were smokers and we may not have captured important views from nurse smokers. However, the prevalence of smoking among nurses in China is below 1%, and we do not believe that views of a nurse who smokes would be substantially different from that of a pediatrician who smokes. Regardless of the limitations, this study draws out some important information and themes for further consideration and action, in relation to promoting SHS exposure reduction in the clinical pediatric setting in China.

## Conclusions

The results of this study suggest that lack of capacity and skills in SHS assessment and counseling has been an obstacle for Chinese pediatricians to provide clinical service on SHS exposure reduction and smoking cessation. The feedback provided by the participants and insights gained from this qualitative study would support the adaptation of an intervention (i.e. the CEASE) to the Chinese pediatric setting following the Chinese healthcare systems and cultural norms, and might contribute to the reduction of SHS exposure of children. Future work should consider development of an intervention, with reference to other evidence based program (i.e. CEASE), for use in the Chinese pediatric setting and test it’s feasibility in addressing SHS exposure of children and parental smoking.

## Electronic supplementary material

Additional file 1: **Typical statements made by participants by key themes.** (DOC 35 KB)
