# Supplementary material for: Secondhand smoke exposure assessment and counseling in the Chinese pediatric setting: a qualitative study
Source: BMC Pediatr. 2014 Oct 15;14:266. doi: 10.1186/1471-2431-14-266 (PMC4287587; doi:10.1186/1471-2431-14-266)
Supplement: Supplementary file 1 — Additional file 1: Typical statements made by participants by key themes. (DOC 35 KB) [file 12887_2014_1188_MOESM1_ESM.doc]

**Additional file 1**

**Typical statements made by participants by key themes**

| **Knowledge of and attitude towards children’s SHS exposure and current practice related to SHS assessment and counseling in Chinese pediatric setting** | **Barriers to SHS exposure reduction counseling in the Chinese pediatric setting** | **Suggestions for overcoming these barriers** |
| --- | --- | --- |
| “Children exposed to tobacco smoke have an increased risk of asthma and respiratory infections. But the link between SHS exposure and some other diseases is uncertain or is not supported by sufficient scientific evidence.” (one pediatrician who smoke and several pediatricians who never smoke)  “I ask about smoking status of parents occasionally and advice the parent to quit or not to smoke around the child. However, I forget doing it regularly.” (One nonsmoker pediatrician)  “I am more concerned about my patients’ (the children’s) illnesses and seldom talk to smoking parents about tobacco control and SHS exposure reduction during routine visits.”( a pediatricians who never smoke)  “I seldom enquired about children’s SHS exposure and parental smoking .I would enquire about it when I treated patients with respiratory diseases, such as asthma.”(many pediatricians and some pediatric nurses)  “It is impossible to routinely address the SHS exposure of children and parental smoking during children’s clinic visits. There are too many patients.” (Several pediatricians who smoke) | “I don’t know how to quit smoking effectively, and I cannot provide effective methods for smoking cessation to smoker parents.”(several pediatricians who smoke)  “I did not know any smoking cessation clinic or quitline in my city.” (The majority of pediatricians and pediatric nurses)  “I have never heard of any cessation clinic or quitline.” (Several pediatricians)  “I have heard of a clinic in my hospital but I have no idea about the program and how to reach them”. (One pediatrician from Nanning Maternal and Child Health Hospital)  “There are too many patients and the parent is not the patient. There is not enough time to spend in counseling smoking parents.”(few pediatricians)  “Counseling smoking parents is too time consuming and will increase my workload. There is no extra time and energy to cope with the extra workload.”(several pediatricians who smoke)  *“*It is useless to counsel smoking parents to quit smoking. Smoking parents would ignore the tobacco control advice. Some smoking parents lack interest in quitting smoking.”(one pediatrician who smoke)  “I am too busy to spend any extra time to enquire about children’s SHS exposure and provide smoking cessation counseling to smoking parents during routine visits.*”*(one smoker pediatrician)  “I read an article which reported that quit smoking advice and counseling is effective to help people quit smoking. The paper also reported that nicotine replacement therapy or other cessation medications may double the success rate of quit smoking.” (One nonsmoker pediatrician).  “As there is no need to record smoking or SHS exposure status of our patients, it is up to the individual to do so. We ask and record the information sometimes, but sometime we are afraid that asking such sensitive question will hamper our relationship with our patients or their family.” (Several nonsmoker pediatrician and nurses, one smoker pediatrician)  “I ask families, mostly father, about smoking in front of the child, if the child has asthma or other respiratory illnesses. It is not required by the pediatrics department, but I do it as my personal interest. Because I myself do not like smoking and I know the harms of smoking to a child’s health*”* (A pediatric nurse) | “The tobacco control work in the United States is outstanding and the support of AAP to test a clinical intervention model in China is encouraging. We may follow the American model and AAP model to promote SHS exposure reduction and smoking cessation in our hospital.” (A hospital administrator)  “Our leaders in the Health Ministry level could focus on this important aspect and should provide us funding to initiate related programs and hiring of additional personnel. Pressure from international bodies, such as AAP, may help them realize the importance”. (One hospital Director)  “SHS exposure reduction intervention ought to be adapted to the Chinese pediatric setting based on Chinese actual conditions. Bringing the United States program to China without modification would not work”(four hospital administrators and several pediatricians)  “we need more training. It will be helpful for me to improve smoking cessation counseling skills. ”we also want more flexibility with scheduling training, which would not occupy our rest time or working time.” “It is better to provide us more written educational materials about SHS exposure reduction and smoking cessation.” “We can obtain training via an online course, which may be more flexible.”(Several pediatricians and pediatric nurses)  “Additional persons should be specially assigned to counsel smoking parents during routine children’s clinic visits, which would not increase pediatricians’ workloads. It may be an acceptable arrangement for pediatric department to implement SHS exposure reduction intervention.” (similar concern was raised by one hospital director and several pediatricians)  “Getting a CME certificate from United States -based university or AAP would encourage many to attend the training program”. (Two pediatricians)  “We have funding difficulties. Otherwise, I shall set up a smoking cessation clinic within the hospital following the hospitals in Hong Kong.” (One non-smoker hospital director)  “I hope international organizations, such as WHO or AAP, could support more training and research activities on tobacco exposure reduction in China, so that our leaders will be encouraged to support large programs.” (Two departmental directors)  “Tobacco use is a sensitive issue and some leaders may smoke themselves. Therefore, ensuring support from the leaders is important to make any system change in the hospital and to train pediatricians in promoting SHS exposure reduction to children.” (Few pediatricians and few nurses) |

**Abbreviations:** AAP– American Academy of Pediatrics; SHS–secondhand smoke；WHO–World Health Organization
